# Trans Fatty Acid Intake Induces Intestinal Inflammation and Impaired Glucose Tolerance

**DOI:** 10.3389/fimmu.2021.669672

**Published:** 2021-04-29

**Authors:** Takuro Okamura, Yoshitaka Hashimoto, Saori Majima, Takafumi Senmaru, Emi Ushigome, Naoko Nakanishi, Mai Asano, Masahiro Yamazaki, Hiroshi Takakuwa, Masahide Hamaguchi, Michiaki Fukui

**Affiliations:** ^1^ Department of Endocrinology and Metabolism, Graduate School of Medical Science, Kyoto Prefectural University of Medicine, Kyoto, Japan; ^2^ Chromatography Mass Spectrometry Sales Department, Life Science and Applied Markets Group, Agilent Technologies, Tokyo, Japan

**Keywords:** innate lymphoid cells, ILC, trans fatty acid, gut microbiota, small intestine

## Abstract

**Background and Aims:**

Many nutritional and epidemiological studies have shown that high consumption of trans fatty acids can cause several adverse effects on human health, including cardiovascular disease, diabetes, and cancer. In the present study, we investigated the effect of trans fatty acids on innate immunity in the gut by observing mice fed with a diet high in trans fatty acids, which have been reported to cause dysbiosis.

**Methods:**

We used C57BL6/J mice and fed them with normal diet (ND) or high-fat, high-sucrose diet (HFHSD) or high-trans fatty acid, high-sucrose diet (HTHSD) for 12 weeks. 16S rRNA gene sequencing was performed on the mice stool samples, in addition to flow cytometry, real-time PCR, and lipidomics analysis of the mice serum and liver samples. RAW264.7 cells were used for the *in vitro* studies.

**Results:**

Mice fed with HTHSD displayed significantly higher blood glucose levels and advanced fatty liver and intestinal inflammation, as compared to mice fed with HFHSD. Furthermore, compared to mice fed with HFHSD, mice fed with HTHSD displayed a significant elevation in the expression of CD36 in the small intestine, along with a reduction in the expression of IL-22. Furthermore, there was a significant increase in the populations of ILC1s and T-bet-positive ILC3s in the lamina propria in mice fed with HTHSD. Finally, the relative abundance of the family *Desulfovibrionaceae*, which belongs to the phylum *Proteobacteria*, was significantly higher in mice fed with HFHSD or HTHSD, than in mice fed with ND; between the HFHSD and HTHSD groups, the abundance was slightly higher in the HTHSD group.

**Conclusions:**

This study revealed that compared to saturated fatty acid intake, trans fatty acid intake significantly exacerbated metabolic diseases such as diabetes and fatty liver.

## Highlights

In this study, we investigated the effect of trans fatty acids on innate immunity in the gut by comparing mice fed with a diet high in trans fatty acids with mice fed with a normal diet or a diet high in saturated fatty acids. We revealed that trans fatty acid intake significantly exacerbated metabolic diseases such as diabetes and fatty liver when compared to saturated fatty acid intake. It has been suggested that this is mainly due to inflammatory modification of the innate immunity, in particular, an increase in the number of intestinal T-bet-positive ILC3s and a significant decrease in the production of IL-22 in the intestine and liver.

## Introduction

Trans fatty acids are a generic term for artificial fatty acids containing trans carbon-carbon double bonds, which are mainly produced in the food production process. Margarine or shortening, a hardened oil produced by hydrogenation, is a typical food with trans fatty acids. Humans now consume trans fatty acids in a way that was not part of our diet in the past. Many nutritional and epidemiological studies have shown that high consumption of trans fatty acids can cause several adverse effects on human health, including cardiovascular disease, diabetes, and cancer (1).

The gut of humans and other mammals contains trillions of microorganisms, which are collectively known as the gut microbiota. The gut microbiota functions as an endocrine organ, helping to shape the intestinal immune response and produce metabolites that are involved in many aspects of the normal host physiology (2). The gut microbiota provides important benefits to the host, especially for metabolic and immune development; thus, homeostasis of the gut microbiota is important for maintaining the health of the host (3). As a result, abnormal changes in the composition and biodiversity of the gut microbiota, known as dysbiosis, can be an important cause of several metabolic syndromes, such as inflammatory bowel disease (IBD), obesity, dyslipidemia, diabetes, heart disease, and cancer (4–8), mediated through chronic inflammation and insulin resistance (4, 8). It is widely accepted that diet is a major factor that regulates the composition of the gut microbiota in humans and mice (7).

In the past decade, a group of lymphocytes, which act in innate immunity and do not express antigen receptors, has been discovered and named innate lymphoid cells (ILCs). Currently, ILCs are classified into three groups: ILC1, ILC2, and ILC3. ILC1s produce IFN-γ to protect against intracellular bacteria and viruses through activation of macrophages. The transcription factor T-bet is involved in the induction of ILC1 differentiation (type 1 immune response). ILC3s produce interleukin (IL)-17 and IL-22, which are involved in the defense against extracellular bacteria and fungi through mobilization of neutrophils, as well as, activation and proliferation of epithelial cells.

In the present study, we hypothesized that trans fatty acids cause dysbiosis and associated immune changes in the intestine. The purpose of this study was to confirm the effect of trans fatty acids on innate immunity in the gut by comparing mice fed with a diet high in trans fatty acids [which have been reported to cause dysbiosis (9)] with mice fed with a normal diet or a diet high in saturated fatty acids.

## Materials and Methods

### Animals

**Figure 1:**
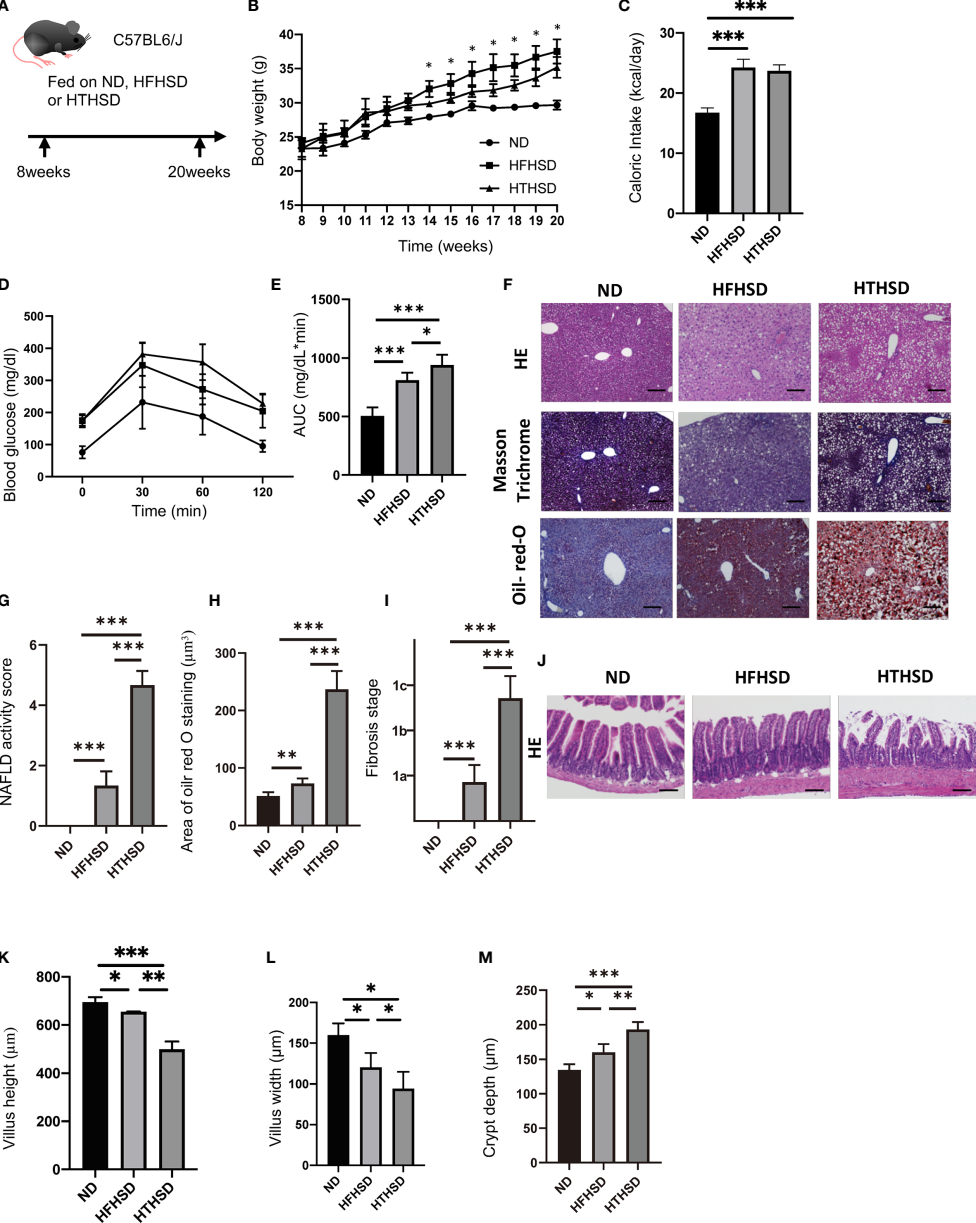
Trans fatty acids significantly worsen glucose tolerance, despite causing only mild weight gain, as compared to saturated fatty acids. **(A)** Eight-week-old C57BL6/J mice were fed with ND, HFHSD, or HTHSD for 12 weeks, starting at 8 weeks of age. The mice were sacrificed when they reached 20 weeks of age. **(B)** Changes in the body weights of the mice (n=6). If the weight of mice fed with HFHSD was significantly higher than that with HTHSD by an unpaired, two-tailed Student’s t test, it was marked with asterisk on top. **(C)** Caloric intake measured at 20 weeks of age (n=6). **(D, E)** When the mice reached 20 weeks of age, an intraperitoneal glucose tolerance test (2 g/kg body weight) was performed, followed by analysis of the area under the curve (n=6). **(F)** Representative liver histology of the mice. **(G)** NAFLD activity score (n=6). **(H)** Oil Red O-stained area (n=6). **(I)** Fibrosis stage (n=6). **(J)** Representative jejunum histology of the mice. **(K)** Villus height (n=6). **(L)** Villus width (n=6). Data are represented as mean ± SD; *p < 0.05, **p < 0.01, ***p < 0.001 using Tukey HSD test. ND, normal diet; HFHSD, high-fat, high-sucrose diet; HTHSD, high-trans fatty acid, high-sucrose diet. **(M)** Crypt depth (n=6).

All experimental procedures were approved by the Committee for Animal Research at the Kyoto Prefectural University of Medicine. Seven-week-old C57BL/6J (wild-type) male mice were purchased from Shimizu Laboratory Supplies (Kyoto, Japan) and housed in a specific pathogen-free controlled environment. We used the littermate mice that were born at the same time at a mouse supply facility. Moreover, we housed weight-matched 10 animals in two cages (5 animals per cage) in each group, experimented with all of them, and took data from a total of six animals, excluding the two larger mice and smaller mice at sacrifice. The mice were fed a normal diet (ND; 345 kcal/100 g, fat kcal 4.6%; CLEA, Japan, Tokyo), or high-fat, high-sucrose diet (HFHSD; 459 kcal/100 g, 20% protein, 40% carbohydrate, and 40% fat, coconut oil; D12327, Research Diets Inc., New Brunswick, NJ, USA), or high-trans fatty acid, high-sucrose diet (HTHSD; 459 kcal/100 g, 20% protein, 40% carbohydrate, and 40% fat, shortening; D18120301, Research Diets Inc.) for 12 weeks, starting at 8 weeks of age. Body weights of the mice were measured every week. When the mice reached 20 weeks of age, they were sacrificed by administration of a combination anesthetic with 0.3 mg/kg medetomidine, 4.0 mg/kg midazolam, and 5.0 mg/kg butorphanol, after an overnight fast (10) (**Figure 1A**).

### Measurement of Caloric Intake

The oral intake of the mice was measured at 20 weeks. Mice were housed individually and weighed food was placed in a trough in each cage. After 24 h, the amount of remaining food was weighed. Caloric intake was calculated by subtracting the final amount of food from the initial amount of food.

### Analytic Procedures for Glucose and Insulin Tolerance Tests

An iPGTT (2 g/kg of body weight) was performed in 20-week-old mice that were made to fast for 16 h. The blood glucose levels were measured from drops of blood at the time-points indicated, using a glucometer (Gultest Neo Alpha; Sanwa Kagaku Kenkyusho, Nagoya, Japan). The results of the iPGTT test were analyzed by measuring the AUC.

### Protocol for Isolation of Mononuclear Cells From Livers and Small Intestines of Mice

To exclude blood contamination in the liver and small intestine, systemic perfusion with heparinized saline was performed before harvesting or washing the liver and small intestinal tissues with phosphate-buffered saline (PBS). The liver was perfused with 20 mL of PBS (pH 7.0) and then harvested. Isolation of hepatic lymphocytes by mechanical dissection was performed using the methods described in previous studies (11). Briefly, a suspension of liver tissue in Roswell Park Memorial Institute (RPMI) 1640 medium containing 20 mL/L fetal bovine serum (FBS, 2%) was centrifuged. The obtained pellet was resuspended in 40% Percoll^®^ solution, layered on top of an equal volume of 60% Percoll^®^ solution and centrifuged, followed by extraction of the middle layer.

Intestinal lamina propria mononuclear cells were isolated according to the instructions of the Lamina Propria Dissociation Kit (130-097-410; Miltenyi Biotec, Germany). Cell pellets were resuspended in 40% Percoll^®^ and added slowly to the upper portion of the centrifuge tubes, which contained 5 mL of 80% Percoll^®^ at the bottom. Lamina propria mononuclear cells were obtained by washing twice with 2% FBS/PBS, post density gradient centrifugation at 420 *× g* for 20 min.

### Tissue Preparation and Flow Cytometry

Stained cells were analyzed on a FACSCanto™ II system and the data were analyzed using FlowJo software version 10 (TreeStar, Ashland, OR, USA). The innate lymphoid cells were gated using methods provided in a previous study (11, 12), with antibodies described in the supplementary file.

### Liver Histology

Liver tissue was obtained and fixed with 10% buffered formaldehyde or embedded in paraffin. Tissue sections were prepared and stained with hematoxylin and eosin (H&E) and Masson’s Trichrome stains. Additionally, the non-alcoholic fatty liver disease (NAFLD) activity score was adopted to assess NAFLD severity, as in our previous study (13), the details for which are provided in the supplementary file.

The liver sections were also subjected to Oil Red O staining. The tissues were fixed in 4% paraformaldehyde overnight at 4°C. Liver tissues, which were frozen in OCT compounds, were cut into 4 μm-thick sections, mounted onto slides, and allowed to dry for 1–2 h. The sections were then rinsed with PBS, pH 7.4. After air drying, the slides were placed in 100% propylene glycol for 2 min and stained with 0.5% Oil Red O solution in propylene glycol for 30 min. The slides were transferred to an 85% propylene glycol solution for 1 min, rinsed in distilled water for two changes, and processed for hematoxylin counterstaining.

Images were captured with a fluorescence microscope BZ-X710 (Keyence, Osaka, Japan), followed by analysis of the Oil Red O-stained area of the liver tissue using ImageJ software.

### Small Intestine Histology

The small intestine tissue was obtained and either fixed with 10% buffered formaldehyde or embedded in paraffin. Tissue sections were prepared and stained with H&E. Images were captured with a fluorescence microscope BZ-X710. The height/width of the villus and crypt depth were analyzed using ImageJ software.

### Gene Expression in Murine Liver and Small Intestine

We used real-time reverse transcription polymerase chain reaction to quantify the mRNA expression levels of *Tnfa, Il6, Il1b*, *Cd36*, *Ccl2*, and *Il22* using the same methods as in our previous study (11), the details for which are provided in the supplementary file. The primer sequence information was provided in the **Supplementary Table**.

### Measurement of Fatty Acid Concentrations in the Liver Tissue and Serum Samples

The fatty acid composition of the murine liver and serum samples was analyzed using gas chromatography-mass spectrometry on an Agilent 7890B/5977B System (Agilent Technologies, Santa Clara, CA, USA). Liver tissue (15 µg) and serum (25 µL) samples were methylated using a Fatty Acid Methylation Kit (Nacalai Tesque, Kyoto, Japan). The final product was loaded onto a Varian capillary column (DB-FATWAX UI; Agilent Technologies). The capillary column used for fatty acid separation was CP-Sil 88 for FAME (100 m × an inner diameter of 0.25 mm × membrane thickness of 0.20 μm, Agilent Technologies). The column temperature was maintained at 100°C for 4 min, increased gradually by 3°C/min to 240°C, and then held there for 7 min. The sample was injected in split mode with a split ratio of 5:1. Each fatty acid methyl ester was detected in the select ion-monitoring mode. All results were normalized to the peak height of the C17:0 internal standard (14).

### Murine Macrophage Cell Culture and Flow Cytometry

Murine macrophage cells (RAW264.7, KAC Co. Ltd., Kyoto, Japan) were seeded into 24-well plates and grown in DMEM supplemented with 10% FBS. RAW264.7 cells were treated with ethanol, 200 μM palmitic acid or elaidic acid for 24 h. Following that, RAW264.7 cells were pre-treated with phorbol myristiric acid (at the indicated concentrations) for 20 min, prior to stimulation with 1 μM ionomycin for cytokine release.

Stained cells were analyzed using FACSCanto™ II system and the data were analyzed using FlowJo software version 10. Antibodies for gating of *il-12-* and *il-1β*-positive cells were the same as those used in our previous study (11, 15), the details for which are provided in the supplementary file.

### Fecal Microbiota Analysis

The fecal samples were collected from the appendix and placed in a cryotube. Immediately afterwards, they were attached to liquid nitrogen for cryopreservation and kept in liquid nitrogen until DNA extraction. Each three fecal samples collected from the appendix of three mice one by one, excluding the one larger mice and smaller mice in a cage of each group. Microbial DNA was extracted from frozen fecal samples using the QIAamp^®^ DNA Stool Mini Kit (Qiagen, Venlo, Netherlands), following the manufacturer’s instructions. The PCR reaction was performed using EF-Taq (SolGent, Korea), with 20 ng of genomic DNA as a template in a 30 µL reaction mixture. The PCR protocol included activation of Taq polymerase at 95°C for 2 min, followed by 35 cycles at 95°C, 55°C, and 72°C for 1 min each, finished with a 10 min step at 72°C. The amplified products were purified using a multiscreen filter plate (Millipore Corp., Billerica, MA, USA). Sequencing was performed using a BigDye™ Terminator v3.1 Cycle Sequencing Kit (Applied Biosystems, Foster City, CA, USA). DNA samples containing the extension products were added to Hi-Di™ formamide (Applied Biosystems). The mixture was incubated at 95°C for 5 min, followed by 5 min on ice, and then analyzed using an ABI Prism 3730xl DNA Analyzer (Applied Biosystems). 16S rRNA sequencing was performed by Macrogen (Seoul, Korea). QIIME version 1.9.1 was used to filter sequences for quality (16). Scores less than 75% and mismatches in the barcode or primers were eliminated from the files. The number of operational taxonomic units (OTUs) was determined using the UCLUST algorithm at 97% similarity (17). Taxonomic assignment of 16S rRNA was performed with the Greengenes core-set-aligned with UCLUST and the UNITE sequence sets for ITS using BLAST (UNITE, 2017). A total of 2,873 OTUs of the 16S rRNA gene and 919 ITS OTUs were used in the subsequent analysis.

The relative abundance of the phenotypic categories of the taxonomic groups was predicted using METAGENassist, a statistical tool for comparative metagenomics (18). Data filtering was based on interquantile range, row normalization by sum, and column normalization based on autoscaling. Differences in microbial communities between three groups were investigated using the phylogeny-based unweighted UniFrac distance metric, and principal coordinate analysis (PCoA) plots and beta-diversity with permutational multivariat analysis of variance (PERMANOVA) test with Tinn-R Gui version 1.19.4.7, R version 1.36 (19). The relative abundance of the bacterial genera between the three groups was evaluated using one-way analysis of variance followed by FDR corrections, and that between two groups was evaluated using the weighted average differences (WAD) method with Tinn-R Gui version 1.19.4.7, R version 1.36 (20), and paired an unpaired, two-tailed Student’s t test with JMP (SAS Institute Inc., Cary, NC, USA). In this WAD method, the genera were ranked by comprehensively assessing higher expression, higher weight, and fold change. WAD was found to be an effective transcriptome analysis. The relative abundance of functional profiles for the gut microbiota in the groups was evaluated using the WAD method and an unpaired, two-tailed Student’s t tests. The top 20 microbial genera in the gut microbiota were determined with the WAD algorithm using R between two groups or small q values evaluated by one-way ANOVA between three groups.

### Statistical Analysis

Differences between two groups were assessed using an unpaired, two-tailed Student’s t test and Mann-Whitney U test for parametric and non-parametric continuous values, respectively. Differences in categorized variables between two groups were assessed using the Pearson’s chi-square test. Differences in continuous variables among more than three groups were assessed using one-way ANOVA. We used Prism software version 8.0 (GraphPad, San Diego, CA, USA). The difference was considered statistically significant at P<0.05.

## Results

### HTHSD Induces Significant Impaired Glucose Tolerance Without Weight Gain

The body weights of mice fed with HFHSD or HTHSD were significantly higher than those of mice fed with ND. Upon comparing HTHSD and HFHSD groups, body weights of the mice were found to be significantly higher in the HFHSD group, from 14 weeks of age (**Figure 1B**). Among the three groups, the caloric intake of the ND group was significantly lower than that of the HFHSD or HTHSD groups, at 20 weeks of age (**Figure 1C**). In the iPGTT, the AUC of blood glucose increased in the following order of the groups - ND, HFHSD, and HTHSD (**Figures 1D, E**).

### HTHSD Aggravates NAFLD and Induces Small Intestinal Inflammation

Both hepatic fat accumulation (**Figures 1F–H**) and liver fibrosis (**Figure 1I** **)** worsened in the following order of the groups - ND, HFHSD, and HTHSD.

Upon histological analysis of the small intestine, villus height and width reduced in the following order of the groups - ND, HFHSD, and HTHSD (**Figures 1J–L**). Conversely, crypt depth increased in the following order of the groups - ND, HFHSD, and HTHSD (**Figure 1M**).

### HTHSD Induces Inflammation and Activation of Fatty Acid Transporter in the Liver and Small Intestine

**Figure 2:**
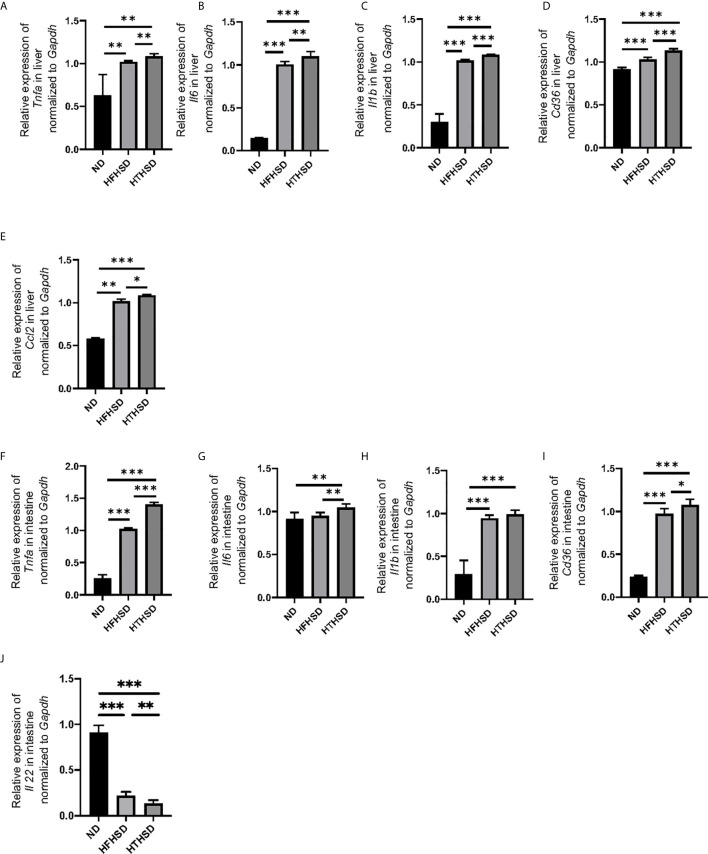
Mice fed with HTHSD displayed significantly elevated inflammation-related gene expression, than mice fed with HTHSD. Gene expression (n=6) in the liver for: **(A)** *Tnfa*, **(B)** *Il6*, **(C)** *Il1b*, **(D)** *Cd36*, and **(E)** *Ccl2*. Gene expression (n=6) in the small intestine for: **(F)** *Tnfa*, **(G)** *Il6*, **(H)** *Il1b*, **(I)** *Cd36*, and **(J)** *Il22*. Data are represented as mean ± SD; *p < 0.05, **p < 0.01, ***p < 0.001 using one-way ANOVA and an unpaired, two-tailed Student’s t test. ND, normal diet; HFHSD, high-fat, high-sucrose diet; HTHSD, high-trans fatty acid, high-sucrose diet.

Next, the relative expression of genes related to inflammation and fatty acid transporters in the liver and small intestine was investigated. Expression levels of *Tnfa, Il6, Il1b*, and *Cd36* in the liver increased in the following order of the groups - ND, HFHSD, and HTHSD (**Figures 2A–D**). Moreover, expression of *Ccl2* increased in the following order of the groups - ND, HFHSD, and HTHSD (**Figure 2E** **)**. Likewise, mice fed with ND, HFHSD, and HTHSD diets displayed increasing expression of *Tnfa* and *Cd36*, with decreasing expression of *Il22* in the small intestine, in that order. Mice fed with HTHSD showed higher expression of *Il-6* in the small intestine than mice fed with ND or HFHSD, and the expression of *Il1b* in mice fed with HFHSD or HTHSD was significantly higher than that with ND, whereas that is not different between mice fed with HFHSD and HTHSD (**Figures 2F–J**). Overall, in both the liver and small intestine, the expression levels of inflammation markers and fatty acid transporters were significantly higher in mice fed with HTHSD, than in those fed with HFHSD.

### HTHSD Induces an Increase in the Number of ILC1s, ILC3s, and M1 Macrophages in the Liver

**Figure 3:**
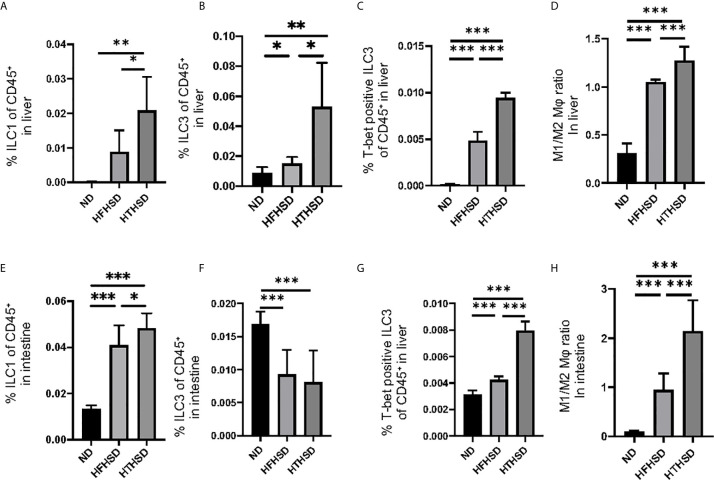
ILC1s, T-bet-positive ILC3s, and M1/M2 macrophages ratio in liver and small intestine in mice fed with HTHSD significantly increased compared. On the other hand, ILC3s in liver in mice fed with HTHSD increased, whereas those in small intestine decreased. **(A)** Ratio of ILC1s to CD45-positive cells in the liver (n=6). **(B)** Ratio of ILC3s to CD45-positive cells in the liver (n=6). **(C)** Ratio of T-bet-positive ILC3s to CD45-positive cells in the liver (n=6). **(D)** Ratio of M1 macrophages to M2 macrophages in the liver (n=6). **(E)** Ratio of ILC1s to CD45-positive cells in the small intestine (n=6). **(F)** Ratio of ILC3s to CD45-positive cells in the small intestine (n=6). **(G)** Ratio of T-bet-positive ILC3s to CD45-positive cells in the liver (n=6). **(H)** Ratio of M1 macrophages to M2 macrophages in the liver (n=6). Data are represented as mean ± SD; *p < 0.05, **p < 0.01, ***p < 0.001 using one-way ANOVA and an unpaired, two-tailed Student’s t test.

Changes in the number of ILCs and macrophages in the liver were examined using flow cytometry (**Supplementary Figures 1, 2**). ILC1s and ILC3s, which have been reported to contribute to the onset of NAFLD [26–27], were significantly higher in the livers of mice fed with HTHSD, as compared to mice fed with ND or HFHSD (ILC1s, ND: *p*=0.009 and HFHSD: *p*=0.029; ILC3s, ND: *p*=0.043 and HFHSD: *p*=0.003) (**Figures 3A, B**). In addition, the number of T-bet-positive ILC3s, which secrete high amounts of IFN-γ [28], were found to increase in the following order of the groups - ND, HFHSD, and HTHSD (**Figure 3C**). There are two main phenotypes of macrophages: M1 pro-inflammatory macrophages and M2 anti-inflammatory macrophages [29]. The M1/M2 macrophage ratio, which indicates an association with liver fibrosis [30], increased in the following order of the groups - ND, HFHSD, and HTHSD (**Figure 3D**).

ILC1s in the lamina propria of the small intestine were higher (both *p*<0.001) in the HFHSD and HTHSD groups than in the ND group; between the HTHSD or HFHSD groups, ILC1 numbers were significantly higher (*p*=0.045) in the HTHSD group (**Figure 3E**). In addition, ILC3s in the lamina propria of the small intestine were lower (both *p*<0.001) in the HFHSD or HTHSD groups than in the ND group; in addition, there was no significant difference (*p*=0.550) between the HFHSD and HTHSD groups (**Figure 3F**).

There was an increase in the number of T-bet-positive ILC3s and the M1/M2 macrophage ratio in the lamina propria of the small intestine, in the following order of the groups - ND, HFHSD, and HTHSD (**Figures 3G, H** **)**.

### Elaidic Acid, Which Increases Upon HTHSD Intake, Induces Inflammation

**Figure 4:**
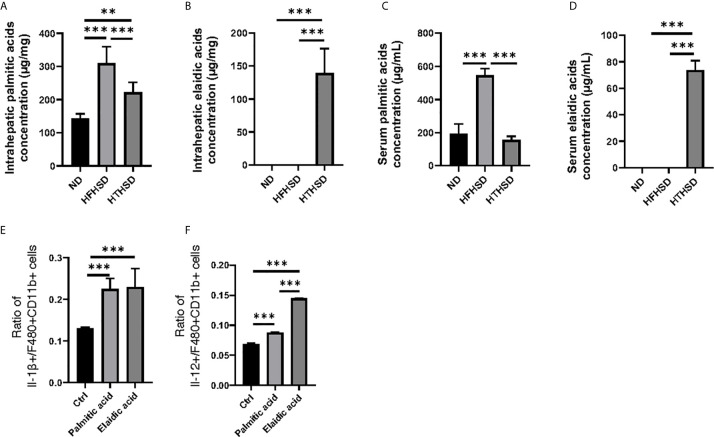
The effect of elaidic acid on IL-1β secretion from macrophages was comparable to that of palmitic acid, and the effect of elaidic acid on IL-12 secretion was significantly stronger. **(A)** Intrahepatic palmitic acid concentration (μg/mg) (n=6). **(B)** Intrahepatic elaidic acid concentration (μg/mg) (n=6). **(C)** Serum palmitic acid concentration (μg/mL) (n=6). **(D)** Serum elaidic acid concentration (μg/mL) (n=6). Data are represented as mean ± SD; **p < 0.01, ***p < 0.001 using one-way ANOVA and an unpaired, two-tailed Student’s t test.

Next, lipidomes in the liver and sera samples were investigated. Among the three groups, palmitic acid concentration was the highest in the liver and sera samples of mice fed with HFHSD, whereas elaidic acid concentration was the highest in the liver and sera samples of mice fed with HTHSD (**Figures 4A–D**). Therefore, ethanol (control), palmitic acid, and elaidic acid were added to RW264.7 cells, a murine macrophage cell line, followed by assessment of the secreted cytokines using multicolor flow cytometric analysis. *IL-1β*- or *IL-12*-positive cells were significantly higher (*p*<0.001) in the groups treated with palmitic acid or elaidic acid, than in the control group. *IL-1β*-positive cells were not significantly different (*p*=0.850) between the palmitic acid- and elaidic acid-treated groups, while *IL-12-*positive cells were significantly higher (*p*<0.001) in the elaidic acid-treated group, than in the palmitic acid-treated group (**Figures 4E, F**).

### HTHSD Diet Induces an Increase in the Abundance of the Phylum Proteobacteria

**Figure 5:**
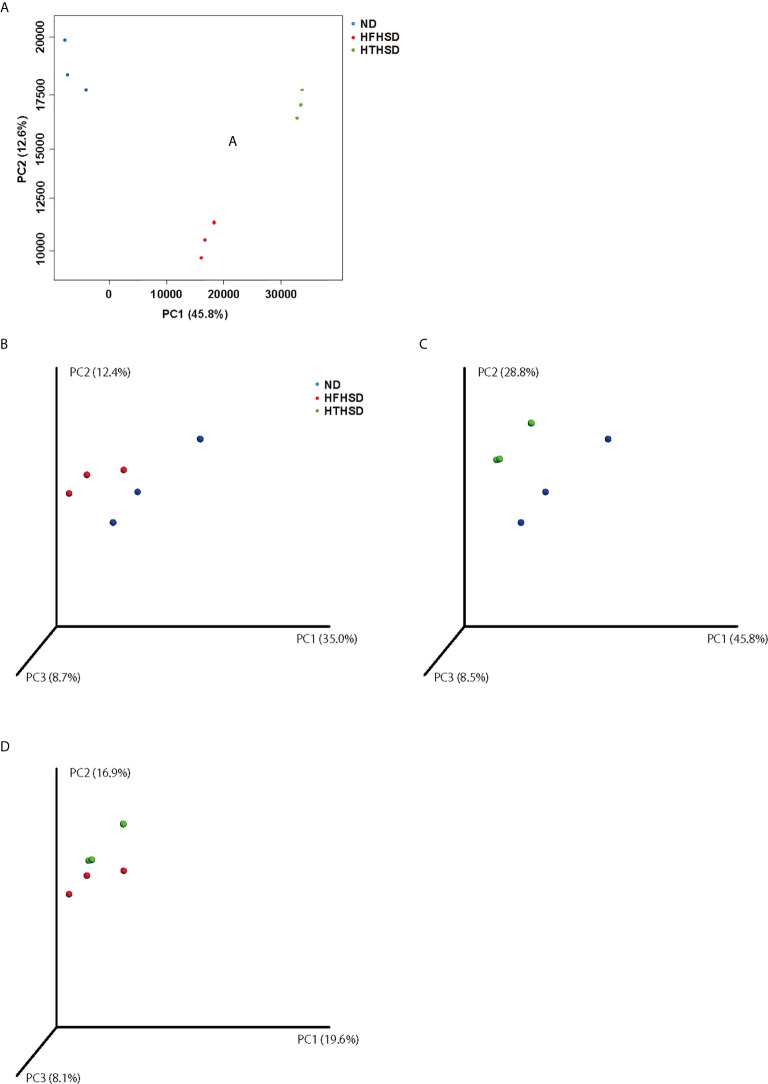
PCoA plots of gut microbiota of mice fed with ND, HFHSD, or HTHSD **(A)** Weighted PCoA plots were shown (n=3). Weighted beta-diversity with PERMANOVA test were shown (n=3). **(B)** ND vs HFHSD (p= 0.010), **(C)** ND vs HTHSD (p= 0.007), and **(D)** HFHSD vs HTHSD (p= 0.055). PCoA: Principal coordinate analysis, PERMANOVA: Permutational multivariat analysis of variance.

**Figure 6:**
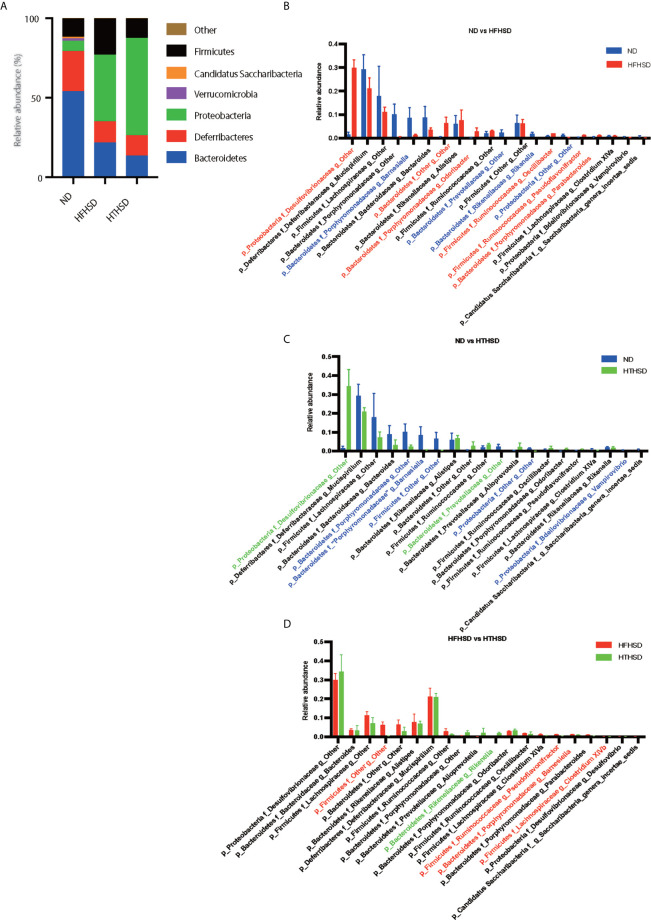
Mice fed with HFHSD or HTHSD displayed significantly higher relative abundance of family *Desulfovibrionaceae* (which belongs to phylum *Proteobacteria)* than mice fed with ND. **(A)** Relative abundance of gut microbiota, by phylum. **(B–D)** Dominant gut microbial genera. The influence of genera of unique gut microbiota in mice fed with ND, HFHSD, or HTHSD was assessed using the weighted average differences method, followed by ranking of the assessed influence of genera from top to bottom. The top 20 gut microbial genera are shown. Relative abundance of gut microbiota in the two groups was compared using an unpaired, two-tailed Student’s t test. Gut microbiota with significantly higher abundance in a group have been indicated in the color of the group. *Red*: ND, *Blue*: HFHSD, *Green*: HTHSD.

Finally, relative abundance of the gut microbiota was investigated using 16s RNA sequencing. PCoA plots were constructed to compare the three groups (**Figure 5A**). Moreover, beta-diversity with PERMANOVA tests were shown in **Figures 5C, D**, and showed notable differences between the ND and HFHSD groups (*p*= 0.010) or the ND and HTHSD groups (*p*= 0.007). On the other hand, there was no significant differences between HFHSD and HTHSD groups (*p*= 0.055) (**Figures 5B–D**). At the phylum level, the relative abundance of *Bacteroidetes* was significantly higher (*p*<0.001) in mice fed with ND (54.1 ± 14.0%) than in mice fed with HFHSD (21.9 ± 6.8%) or HTHSD (13.5 ± 10.3%); there was no significant difference (*p*=0.301) between the HFHSD and HTHSD groups (**Figure 6A**). Likewise, the relative abundance of *Deferribacteres* was significantly higher (*p*<0.001) in mice fed with ND (25.1 ± 5.9%) than in mice fed with HFHSD (13.1 ± 2.8%) or HTHSD (12.9 ± 2.5%); there was no significant difference (*p*=0.931) between the HFHSD and HTHSD groups. On the contrary, the relative abundance of *Proteobacteria* was significantly higher in mice fed with ND (6.5 ± 2.8%), HFHSD (42.0 ± 3.6%), and HTHSD (61.3 ± 9.9%) (all *p*<0.001) (**Figure 6A**). At the genus level, the top 20 species were ranked using the WAD method. Upon comparing mice fed with ND and HFHSD, the relative abundance of the family *Desulfovibrionaceae*, which belongs to the phylum *Proteobacteria* (29.7 ± 3.6% vs. 1.4 ± 1.1%, *p*=0.005), genus *Odoribacte*, genus *Oscillibacte*, genus *Pseudoflavonifractor*, and genus *Parabacteroides* was significantly higher in mice fed with HFHSD, than in mice fed with ND. On the contrary, genus *Barnesiella*, family *Prevotellaceae*, and genus *Rikenella* were significantly lower in mice fed with HFHSD than in those fed with ND (**Figure 6B**). Upon comparing mice fed with ND and HTHSD, the relative abundance of the family *Desulfovibrionaceae* was significantly higher in mice fed with HTHSD than in mice fed with ND (3.3 ± 0.9% vs. 1.9 ± 0.9%, *p*=0.009). In contrast, the relative abundance of genus *Barnesiella*, family *Prevotellaceae*, and genus *Vampirovibrio* was significantly lower in mice fed with HFHSD, than in mice fed with ND (**Figure 6C**). On the other hand, there were no big differences in the relative abundance of genera between mice fed with HTHSD or HFHSD. However, the relative abundance of genus *Rikenella*, which belongs to the family *Rikenellaceae*, was significantly higher in mice fed with HTHSD, than in mice fed with HFHSD (1.7 ± 0.8% vs. 0.0 ± 0.0%, *p*=0.018). The relative abundance of the family *Desulfovibrionaceae* was higher in mice fed with HTHSD, than in mice fed with HFHSD, although the difference was not statistically significant. Moreover, the relative abundance of phylum *Firmicutes*, genus *Psudoflavonifractor*, genus *Barnesiella*, and genus *Clostridium XIVb* was significantly lower in mice fed with HTHSD, than in mice fed with HFHSD (**Figure 6D**). Furthermore, in one-way ANOVA, the similar results were obtained (**Supplementary Figure 3**).

## Discussion

The present study revealed that, as compared to saturated fatty acids, trans fatty acids induced more intestinal inflammation and resulted in significantly impaired glucose tolerance with increased hepatic fat accumulation and progression of liver fibrosis. Previous studies have shown that trans fatty acids are risk factors for cardiovascular disease, diabetes, and cancer (1, 21, 22). This study provides a basis for this increased risk.

Some possible explanations for this observation are listed below. Secretion of *Il-6*, a pro-inflammatory cytokine, has been found to be higher in mice fed with an elaidic acid-rich diet, as compared to mice fed with a PUFA-rich diet (23). In addition, activation of *NF-κB* and *Tnfa*, as well as expression of *Ccl2*, osteopontin, and macrophage markers in the liver have been found to be elevated in mice fed with a trans fatty acid-rich diet (24–26). Hirata et al. (27) showed that, as compared to both control and oleic acid, 12 h of incubation with 0.2 mM elaidic acid induced caspase 3 cleavage and promoted apoptotic cell death in RAW264.7 macrophages, mediated *via* over-activation of the apoptosis signal-regulated kinase 1-p38 mitogen-activated protein kinase pathway. Ge et al. (9) reported that a diet enriched with trans fatty acids induced lipid deposition in small intestinal epithelial cells and destruction of the small intestinal epithelium. In addition, a significant increase in the expression levels of Cxcl12, Cxcl14, and Cxcr4 has been observed in the small intestine of mice fed with trans fatty acids. In the present study, the number of M1 macrophages and ILC1 in the mucous membranes of the liver and small intestine were predominantly increased in the trans-fatty acid-fed mice, and the expression levels of inflammation-related genes, such as *Tnfa*, *Il6*, and *Il1b*, were also predominantly increased. In addition, the pathological images of small intestine showed that the HFHSD group had a significant decrease in the height and width of the villi and an increase in the depth of the crypt, indicating inflammation of the small intestinal mucosa.

Ge et al. (9) also reported that trans fatty acid-rich diet caused a decrease in the relative abundance of the phylum *Bacteroidetes*, along with an increase in the phylum *Proteobacteria* and the family *Desulfovibrionaceae*, which are gram-negative sulfate-reducing bacteria found to be significantly abundant in obese and metabolically-impaired mice (28, 29). The results of the present study are consistent with those of the above studies. Increased relative abundance of *Desulfovibrionaceae* and the resulting excessive hydrogen sulfide (H_2_S) production may contribute to IBDs and inflammation-related bowel diseases, such as colorectal cancer (30). H_2_S enhances the breakdown of the mucus barrier by decreasing the disulfide bonds in the mucus network, thus leading to an increase in the permeability of the mucus layer (31). Rupture of the mucus barrier allows toxins and bacteria to come into close proximity to the colonic epithelium, causing inflammation, and ultimately, contributing to the development of IBD and colorectal cancer (31). Furthermore, failure of the mucus barrier has been reported to alter the innate immunity of the intestinal tract. In particular, the number of ILC3s, which are major regulators of inflammation and infection at mucosal barriers, are altered by failure of the mucus barrier (32). ILC3-derived IL-22 plays an important role in promoting STAT3-dependent expression of antimicrobial peptides and maintenance of the intestinal epithelial barrier function (33–35). Conversely, loss of ILC3s is associated with reduced expression of IL-22 and lower levels of antimicrobial peptides expressed by the intestinal epithelial cells. In the present study, intake of HFHSD or HTHSD increased the abundance of the family *Desulfovibrionaceae* in the gut microbiota and decreased the number of ILC3s in the lamina propria of the small intestine. Additionally, the increased abundance of the family *Desulfovibrionaceae* has been reported to be associated with increased fat absorption (36, 37). In our study, expression of Cd36, a transporter of long-chain fatty acids, in the small intestine was significantly higher in mice fed with HFHSD or HTHSD, than in those fed with ND; between the HTHSD and HFHSD groups, HTHSD displayed higher expression. At the same time, mice fed with HTHSD displayed higher abundance of the family *Desulfovibrionaceae*, as compared to those fed with HFHSD, although the difference was not significant. Moreover, the relative abundance of the genus *Rikenella*, which belongs to the family *Rikenellaceae*, was significantly higher in mice fed with HTHSD than in those fed with HFHSD. Several previous studies have reported that the abundance of the family *Rikenellaceae* increases upon intake of a high-fat diet (38), and this increase is related to the loss of gut barrier function (39).

In summary, the failure of the mucus barrier was more severe in the mice fed with HTHSD, than in the mice fed with HFHSD, due to an increase in the relative abundance of the family *Desulfovibrionaceae* and genus *Rikenella*, which might be responsible for causing various metabolic disorders.

The increased ratio of ILC1s and decreased ratio of RORγt-positive ILC3s in the small intestine were observed to a similar degree in both mice fed with HFHSD and HTHSD, whereas the ratio of T-bet-positive ILC3s in the small intestine was significantly higher in mice fed with HTHSD, than in mice fed with HFHSD. There is some evidence that ILCs can exhibit functional plasticity in response to environmental cues. The function of ILC3s has been shown to vary with the expression of the transcription factors RORγt and T-bet (40). Stimulation of cytokines such as IL-12 and IL-18 increases the number of ex-RORγt-positive ILC3s, which are characterized to be T-bet positive, and decreases the number of RORγt-positive ILC3s, indicating that ILC3s are able to respond to environmental cues. It has been reported that ex-RORγt-positive ILC3s have the ability to produce IFNγ and reduce the production of IL-17 and IL-22 (41). Thus, ex-RORγt-positive ILC3s exhibit functions similar to those of ILC1s. In addition, in cell experiments using RAW264.7, elaidic acid, which was elevated in the liver and serum samples of mice fed with HTHSD, did not significantly increase the ratio of IL-1β-positive cells, as compared to palmitic acid, which was elevated in the liver and serum samples of mice fed with HFHSD. IL-1β has been reported to accelerate the dedifferentiation of ILC1s to ILC3s (42). It has been suggested that there is a compensatory increase in IL-1β secretion, to increase the number of ILC3s, which attenuates intestinal mucosal inflammation. On the other hand, as compared to cells administered with palmitic acid, the ratio of IL-12-positive cells was significantly higher in cells administered with elaidic acid. Therefore, it is thought that, as compared to palmitic acid stimulation, IL-12 secreted by elaidic acid stimulation of macrophages increases the number of ILC1s (43) and differentiates ILC3s into ex-RORγt-positive ILC3s to a greater extent. In this study, intake of HTHSD might have decreased the production of IL22s in intestinal epithelial tissues by increasing the number of ex-RORγt-positive ILC3s, which strongly express T-bet and lack the ability to produce IL-22, thus reducing the maintenance of antimicrobial peptides and intestinal epithelial barrier function. On the other hand, the number of ILC3s in the liver was significantly higher in the HFHSD or HTHSD groups, than in the ND group, a trend opposite to that observed in the case of ILC3s in the lamina propria of the small intestine. In our previous study, we found that HFD treatment caused a compensatory increase in the number of ILC3s, to reduce inflammation in the liver (11). In the present study, inflammation in the small intestinal mucosa was the primary and important change, while an increase in the number of ILC3s in the liver was thought to be compensatory, due to the associated hepatitis and liver fibrosis.

As limitation of this study, we do not have the data of the microbiome longitudinally i.e., at baseline and at several timepoint. This data could have clarified the effect of HFSHSD and HTHSD in changing gut microbiota.

Taken together, the present study revealed that the intake of trans-fatty acids and saturated fatty acids caused dysbiosis and associated immune changes in the intestine, and significantly aggravated metabolic diseases such as diabetes and fatty live when compared with the intake of normal diet, and this was more pronounced for trans-fatty acids. In addition, trans-fatty acids strongly activate the differentiation of RORgt-positive ILC3 into T-bet-positive ILC3 by promoting the secretion of IL-12 from macrophages more strongly than saturated fatty acids.

## Data Availability Statement

The datasets presented in this study can be found in online repositories. The names of the repository/repositories and accession number(s) can be found below: https://doi.org/10.6084/m9.figshare.14370035.v1.

## Ethics Statement

The animal study was reviewed and approved by The Committee for Animal Research at the Kyoto Prefectural University of Medicine.

## Author Contributions

TO originated and designed the study, researched the data, and wrote the manuscript. YH and MH originated and designed the study, researched the data, and reviewed the manuscript. SM, TS, EU, NN, MA, and MY researched the data and contributed to the discussion. HT provided technical cooperation. MF originated and designed the study, researched the data, and reviewed and edited the manuscript. MF is the guarantor of this work and, as such, had full access to all of the data in the study and takes responsibility for the integrity of the data and the accuracy of the data analysis. All authors contributed to the article and approved the submitted version.

## Funding

This work was supported by The Japan Food Chemical Research Foundation.

## Conflict of Interest

HT was employed by Agilent Technologies. YH has received grants from Asahi Kasei Pharma, personal fees from Daiichi Sankyo Co., Ltd., personal fees from Mitsubishi Tanabe Pharma Corp., personal fees from Sanofi K.K., personal fees from Novo Nordisk Pharma Ltd., outside the submitted work. TS has received personal fees from Ono Pharma Co., Ltd., Mitsubishi Tanabe Pharma Co, Astellas Pharma Inc., Kyowa Hakko Kirin Co., Ltd., Sanofi K.K., MSD K.K., Kowa Pharma Co., Ltd., Taisho Toyama Pharma Co., Ltd., Takeda Pharma Co., Ltd., Kissei Pharma Co., Ltd., Novo Nordisk Pharma Ltd., Eli Lilly Japan K.K. outside the submitted work. MH has received grants from Asahi Kasei Pharma, Nippon Boehringer Ingelheim Co., Ltd., Mitsubishi Tanabe Pharma Corporation, Daiichi Sankyo Co., Ltd., Sanofi K.K., Takeda Pharmaceutical Company Limited, Astellas Pharma Inc., Kyowa Kirin Co., Ltd., Sumitomo Dainippon Pharma Co., Ltd., Novo Nordisk Pharma Ltd., and Eli Lilly Japan K.K., outside the submitted work. MA received personal fees from Novo Nordisk Pharma Ltd., Abbott Japan Co., Ltd., AstraZeneca plc, Kowa Pharmaceutical Co., Ltd., Ono Pharmaceutical Co., Ltd., Takeda Pharmaceutical Co., Ltd., outside the submitted work. MY reports personal fees from MSD K.K., Sumitomo Dainippon Pharma Co., Ltd., Kowa Company, Limited, AstraZeneca PLC, Takeda Pharmaceutical Company Limited, Kyowa Hakko Kirin Co., Ltd., Daiichi Sankyo Co., Ltd., Kowa Pharmaceutical Co., Ltd., Ono Pharma Co., Ltd., outside the submitted work. MF has received grants from Nippon Boehringer Ingelheim Co., Ltd., Kissei Pharma Co., Ltd., Mitsubishi Tanabe Pharma Co, Daiichi Sankyo Co., Ltd., Sanofi K.K., Takeda Pharma Co., Ltd., Astellas Pharma Inc., MSD K.K., Kyowa Hakko Kirin Co., Ltd., Sumitomo Dainippon Pharma Co., Ltd., Kowa Pharmaceutical Co., Ltd., Novo Nordisk Pharma Ltd., Ono Pharma Co., Ltd., Sanwa Kagaku Kenkyusho Co., Ltd. Eli Lilly Japan K.K., Taisho Pharma Co., Ltd., Terumo Co., Teijin Pharma Ltd., Nippon Chemiphar Co., Ltd., and Johnson & Johnson K.K. Medical Co., Abbott Japan Co., Ltd., and received personal fees from Nippon Boehringer Ingelheim Co., Ltd., Kissei Pharma Co., Ltd., Mitsubishi Tanabe Pharma Corp., Daiichi Sankyo Co., Ltd., Sanofi K.K., Takeda Pharma Co., Ltd., Astellas Pharma Inc., MSD K.K., Kyowa Kirin Co., Ltd., Sumitomo Dainippon Pharma Co., Ltd., Kowa Pharma Co., Ltd., Novo Nordisk Pharma Ltd., Ono Pharma Co., Ltd., Sanwa Kagaku Kenkyusho Co., Ltd., Eli Lilly Japan K.K., Taisho Pharma Co., Ltd., Bayer Yakuhin, Ltd., AstraZeneca K.K., Mochida Pharma Co., Ltd., Abbott Japan Co., Ltd., Medtronic Japan Co., Ltd., Arkley Inc., Teijin Pharma Ltd. and Nipro Cor., outside the submitted work.

The remaining authors declare that the research was conducted in the absence of any commercial or financial relationships that could be construed as a potential conflict of interest.
