# Supplementary material for: Trans Fatty Acid Intake Induces Intestinal Inflammation and Impaired Glucose Tolerance
Source: Front Immunol. 2021 Apr 29;12:669672. doi: 10.3389/fimmu.2021.669672 (PMC8117213; doi:10.3389/fimmu.2021.669672)
Supplement: Supplementary file 1 [file DataSheet_1.pdf]

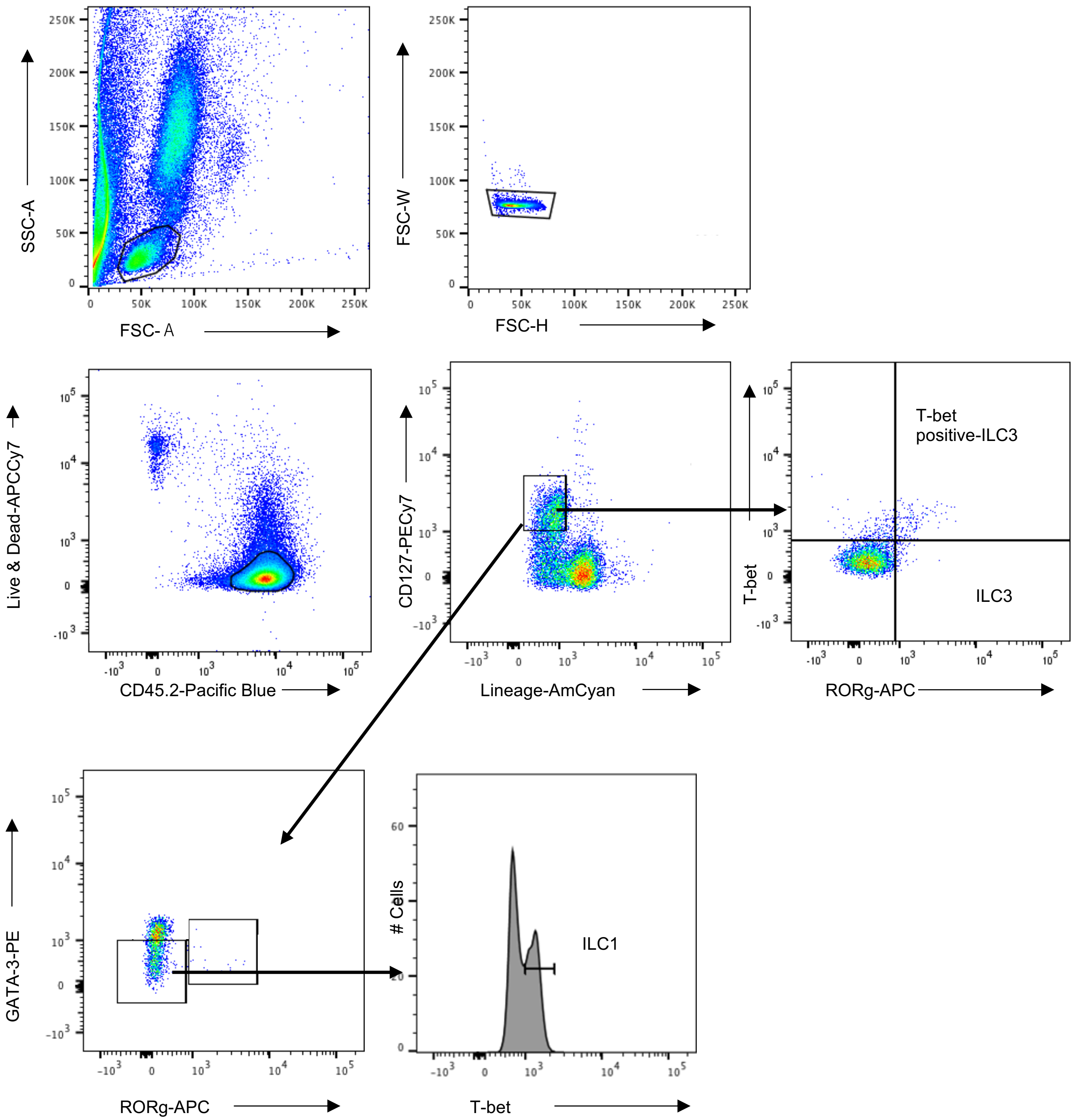

Supplementary Figure 1. Strategy for innate lymphoid cells (ILCs)

Representative flow cytometry plots of liver CD45+ Live & Dead- Lin- CD127+ RORg- GATA-3- T-bet+ ILC1s, CD45+ Live & Dead- Lin- CD127+ RORg+ T-bet- ILC3s and CD45+ Live & Dead- Lin- CD127+ RORg+ T-bet+ T-bet positive ILC3s in each group at 20-weeks of age.

Supplementary Fig. 1

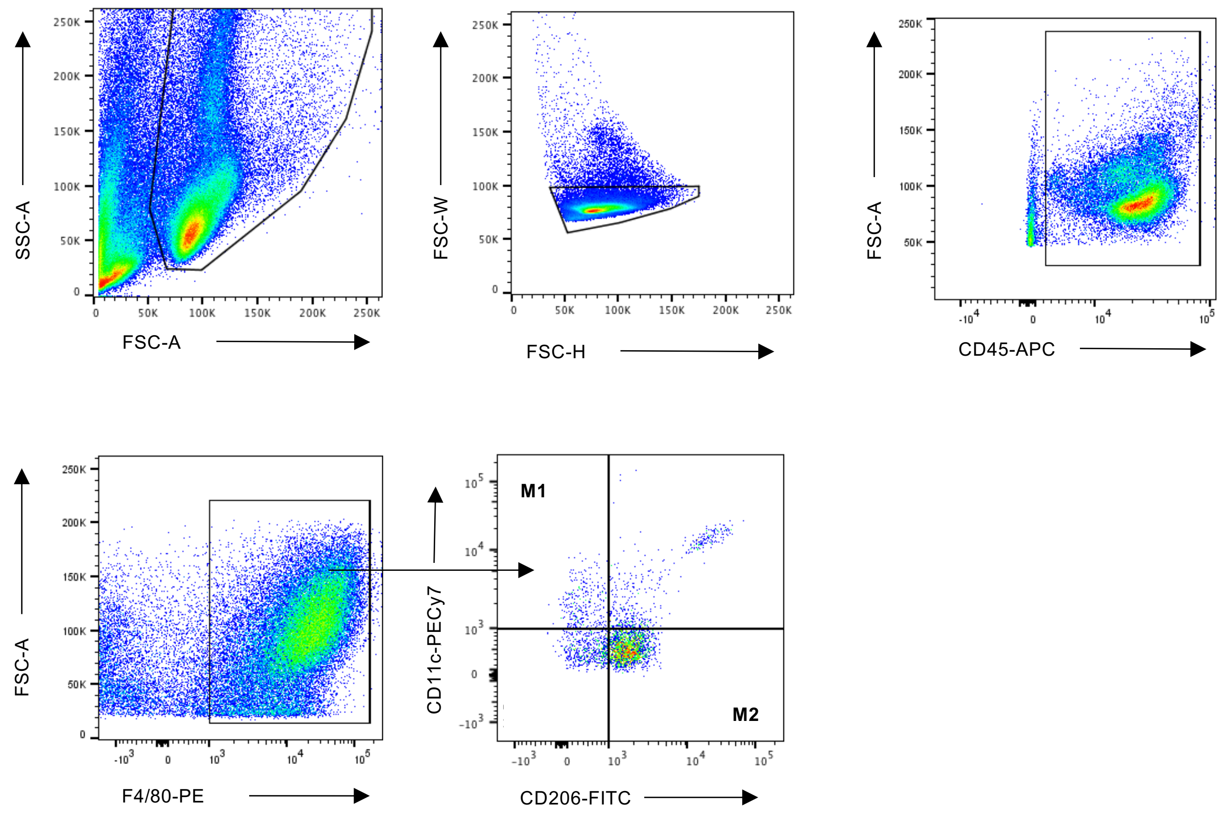

Supplementary Figure 2. Strategy for macrophages

Representative flow cytometry plots of WAT CD45+ F4/80+ CD206- CD11c+ M1 macrophages and CD45+ F4/80+ CD206+ CD11c- M2 macrophages in each group at 20 weeks of age.

Supplementary Fig. 2

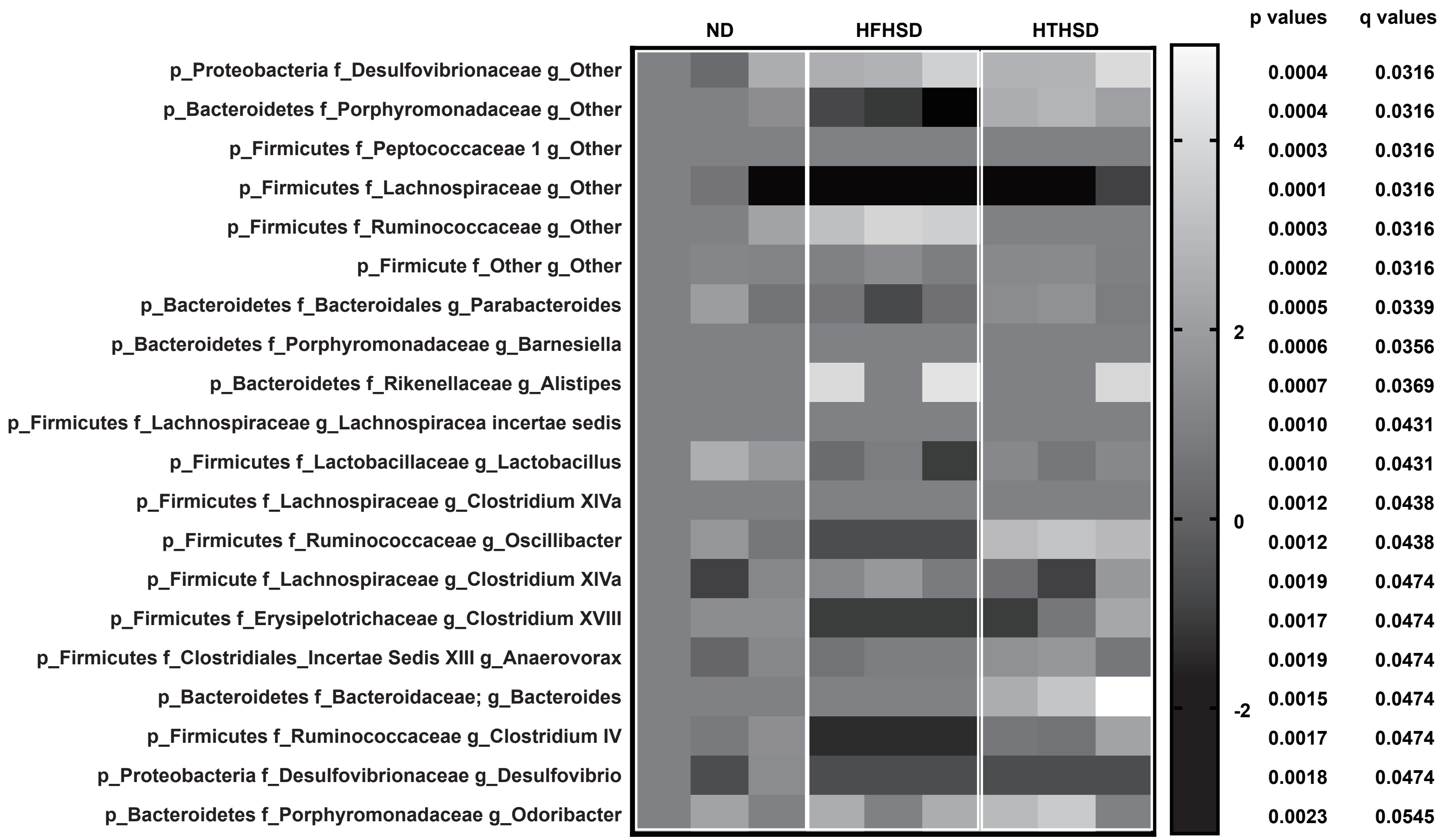

Supplementary Figure 3. Heat map of the top 20 gut microbial genera

The top 20 gut microbial genera are shown. Relative abundance of gut microbiota in the three groups was compared using one-way ANOVA.
