# Supplementary material for: Trans Fatty Acid Intake Induces Intestinal Inflammation and Impaired Glucose Tolerance
Source: Front Immunol. 2021 Apr 29;12:669672. doi: 10.3389/fimmu.2021.669672 (PMC8117213; doi:10.3389/fimmu.2021.669672)
Supplement: Supplementary file 2 [file DataSheet_2.docx]

**Materials and methods**

*Tissue preparation and flow cytometry*

Biotin-CD3e (100304; clone: 145-2C11; 1/200; eBioscience, San Diego, CA, USA), Biotin- CD45R/B220 (103204; clone: RA3–6B2; 1/200; eBioscience), Biotin-Gr-1 (108404; clone: RB6-8C5; 1/200; eBioscience), Biotin-CD11c (117304; clone: N418; 1/200; eBioscience), Biotin-CD11b (101204; clone: M1/70; 1/200; eBioscience), Biotin-Ter119 (116204; clone: TER-119; 1/200; eBioscience), Biotin-FceRIa (134304; clone: MAR-1; 1/200; eBioscience), FITC-Streptavidin (405202; 1/500; eBioscience), PE-Cy7-CD127 (135014; clone: A7R34; 1/100; eBioscience), Pacific Blue-CD45 (103116; clone: 30-F11; 1/100; eBioscience), PE -GATA-3 (clone TWAJ, 1/50; eBioscience), APC -RORγ (clone AFKJS-9, 1/50, eBioscience), and Fixable Viability Dye eFluor 780 (1/400; eBioscience) (16)(17). Additionally, we used the following antibodies for gating of M1 and M2 macrophages: APC- CD45.2 (17045482; clone: 104, 1/50; eBioscience), PE-F4/80 (12480182; clone: BM8, 1/50, eBioscience), APC-Cy7-CD11b (47011282; clone: M1/70, 1/50; eBioscience), FITC-CD206 (MA516870; clone: MR5D3, 1/50, eBioscience), and PE-Cy7-CD11c (25011482; clone: N418, 1/50, eBioscience).

*Liver histology*

NAFLD activity score, which is a well-known standard, used for assessing NASH severity and measuring changes in NAFLD. A trained hepatopathologist, who was masked to the experimental conditions, examined all the sections for steatosis, lobular inflammation, hepatocyte ballooning, and fibrosis, according to the NAFLD activity score (NAS) (19). Briefly, the scoring system comprised 14 histological features, four of which were evaluated semi-quantitatively: steatosis (0–3), lobular inflammation (0–2), hepatocellular ballooning (0–2), and fibrosis (0–4). In addition, to assess the fibrosis, Stage 1 was classified as 1A for mild peri-central perisinusoidal fibrosis, 1B for moderate or greater perisinusoidal fibrosis, and 1C for fibrosis in the portal region or peri-portal vein. Stage 2 was classified for perisinusoidal and periportal fibrosis, Stage 3 was classified for bridging fibrosis, and Stage 4 was classified for liver cirrhosis.

*Gene expression in murine liver and small intestine*

The liver and small intestine tissues of fasting mice were resected and immediately frozen using liquid nitrogen. The liver and small intestine tissues were homogenized in ice-cold QIAzol Lysis reagent (Qiagen, Hilden, Germany), and total RNA was isolated according to the manufacturer's instructions. Total RNA (0.5 μg) was reverse-transcribed using a High-Capacity cDNA Reverse Transcription Kit (Applied Biosystems, Foster City, CA, USA) for first-strand cDNA synthesis, using an oligonucleotide dT primer and random hexamer priming, according to the manufacturer's recommendations. The reverse transcription reaction was performed for 120 min at 37°C and the inactivation of reverse transcription was performed for 5 min at 85°C. The RT-PCR was performed using TaqMan Fast Advanced Master Mix (Applied Biosystems), according to the manufacturer's instructions. The following PCR conditions were used: 1 cycle for 2 min at 50°C and 20 s at 95°C, followed by 40 cycles for 1 s at 95°C, and 20 s at 60°C.

The relative expression levels of each targeted gene were normalized to *Gapdh* threshold cycle (CT) values and quantified using the comparative threshold cycle 2−ΔΔCT method. Signals from C57BL6/J mice fed ND were assigned a relative value of 1.0. Six mice from each group were examined, and an RT-PCR was run in triplicate for each sample.

*Murine macrophage cell culture and flow cytometry*

We used the following antibodies for gating of *il-12* and *il-1β* positive cells: PE-Cy-7-CD45 (103113; clone: 30-F11; 1/100; BioLegend, San Diego, CA, USA), Pacific Blue-F4/80 (123123; clone: BM8; 1/100; BioLegend), PerCP-Cy5.5-CD11b(101227; clone: M1/70; 1/100; BioLegend), PE-IL-1β (17711480; clone: NJTEN3; 1/50; Invitrogen, Carlsbad, CA, USA), and FITC-IL-12 (560564; clone: C15.6; 1/50, Biosciences.

Supplementary Table. Primer sequences of mouse for RT-PCR

| Primer name | Forward | Reverse |
| --- | --- | --- |
| TNFα | ATGGATCTCAAAGACAACCAACTAG | ACGGCAGAGAGGAGGTTGACTT |
| IL-6 | AACCACGGCCTTCCCTACTT | TCTGTTGGGAGTGGTATCCTCTGT |
| IL-1β | TCGTGCTGTCGGACCCATAT | GGTTCTCCTTGTACAAAGCTCATG |
| CD36 | GCTTGCAACTGTCAGCACAT | GCCTTGCTGTAGCCAAGAAC |
| CCL2 | GCTACAAGAGGATCACCAGCAG | GTCTGGACCCATTCCTTCTTGG |
| IL-22 | ATGAGTTTTTCCCTTATGGGGAC | GCTGGAAGTTGGACACCTCAA |
| GAPDH | TGAGCAAGAGAGGCCCTATC | AGGCCCCTCCTGTTATTATG |
